# Motor recovery of hemiparetic leg by improvement of limb-kinetic apraxia in a chronic patient with traumatic brain injury: A case report

**DOI:** 10.1097/MD.0000000000020144

**Published:** 2020-05-08

**Authors:** Eun Bi Choi, Jun Young Kim, Sung Ho Jang

**Affiliations:** Department of Physical Medicine and Rehabilitation, College of Medicine, Yeungnam University, Namku, Daegu, Republic of Korea.

**Keywords:** apraxia, diffusion tensor imaging, diffusion tensor tractography, limb-kinetic apraxia, motor recovery, stroke

## Abstract

**Rationale:**

Limb-kinetic apraxia (LKA), a kind of apraxia, means the inability to perform precise and voluntary movements of extremities resulting from injury of the premotor cortex (PMC) or the corticofugal tract (CFT) from the PMC. Diagnosis of LKA is made by observation of movements without specific assessment tools.

**Patient concerns:**

A 44-year-old male underwent conservative management for traumatic intracerebral hemorrhage in the left basal ganglia and subarachnoid hemorrhage due to a pedestrian-car crash. When he was admitted to the rehabilitation department of a university hospital after 41 months after onset, he presented with right hemiparesis (Medical Research Council (MRC): shoulder abductor; 3, elbow flexor; 3, finger extensor; 0, hip flexor; 2- [range: 30°], knee extensor; 1 and ankle dorsiflexor; 3-). In addition, he exhibited slow, clumsy, and mutilated movements when performing movements of his right ankle.

**Diagnoses:**

The patient was diagnosed as traumatic brain injury (TBI).

**Interventions:**

Clinical assessments and DTI were performed at 41 and 44 months after onset. During three months, rehabilitative therapy was performed including dopaminergic drugs (pramipexole 2.5 mg, ropinirole 2.5 mg, and amantadine 300 mg, and carbidopa/levodopa 75 mg/750 mg).

**Outcomes:**

The right leg weakness slowly recovered during 3 months, until 44 months after the initial injury (MRC: shoulder abductor, 3; elbow flexor, 3; finger extensor, 0; hip flexor, 3; knee extensor, 3; and ankle dorsiflexor, 3+). The fiber number of the right corticospinal tract (CST) was decreased on 44-month diffusion tensor tractography (DTT) (1319) compared with 41-month DTT (1470) and the left CST was not reconstructed on both DTTs. The fiber number of both CRTs were decreased on 44-month DTT (right: 1547, left: 698) than 41-month DTT (right: 3161, left: 1222).

**Lessons:**

A chronic patient with TBI showed motor recovery of the hemiparetic leg by improvement of LKA after rehabilitation. This results have important implications for neurorehabilitation.

## 1 Introduction

Elucidation of the motor recovery mechanisms in patients with motor weakness due to brain injury is clinically important because such knowledge could provide the scientific basis for neurorehabilitation. Several following mechanisms of motor recovery in patients with brain injury have been proposed: the ipsilateral motor pathway from the unaffected motor cortex to the affected hand, perilesional reorganization, contribution of the secondary motor area, restoration of the normal integrity of the injured neural tract for motor function, aberrant pathway of the injured neural tract for motor function, and contribution of transcallosal fibers which connected to the neural tract for motor function.^[1–4]^ Except for the above those, a few case studies have reported on the motor recovery by improvement of limb-kinetic apraxia (LKA) in patients with brain injury.^[5–7]^ However, it has not clearly clarified.

Apraxia is defined as an inability to correctly carry out purposeful skilled movements when this deficit is not caused by elemental motor for sensory deficits, abnormal involuntary movements, or cognitive disorders.^[8]^ LKA, a kind of apraxia, means the inability to perform precise and voluntary movements of extremities resulting from injury of the premotor cortex (PMC) or the corticofugal tract (CFT) from the PMC.^[8–13]^ Diagnosis of LKA is important because LKA can be improved by the administration of the dopaminergic drugs.^[5–7]^ However, precise diagnosis of LKA is difficult because it is made by clinical observation of movements without specific assessment tools.^[10–12]^ Denes et al (1998)^[12]^ described the following criteria to classify a movement error of LKA: awkwardness and clumsiness of the movement in the absence of elementary motor and sensory deficit, ataxia, and in the presence of normal muscle tone; absence of voluntary automatic dissociation (ie, the motor impairment) should equally affect both movements performed in daily living activities and those performed in the testing sessions; the motor impairment should affect equally the execution of both symbolic and meaningless gestures; constancy of the impairment throughout the various observations; and normal conceptual knowledge of the movements.

In the present study, we report on a chronic patient with traumatic brain injury (TBI) who showed motor recovery of the hemiparetic leg by improvement of LKA after rehabilitation.

## 2 Case report

**Figure 1:**
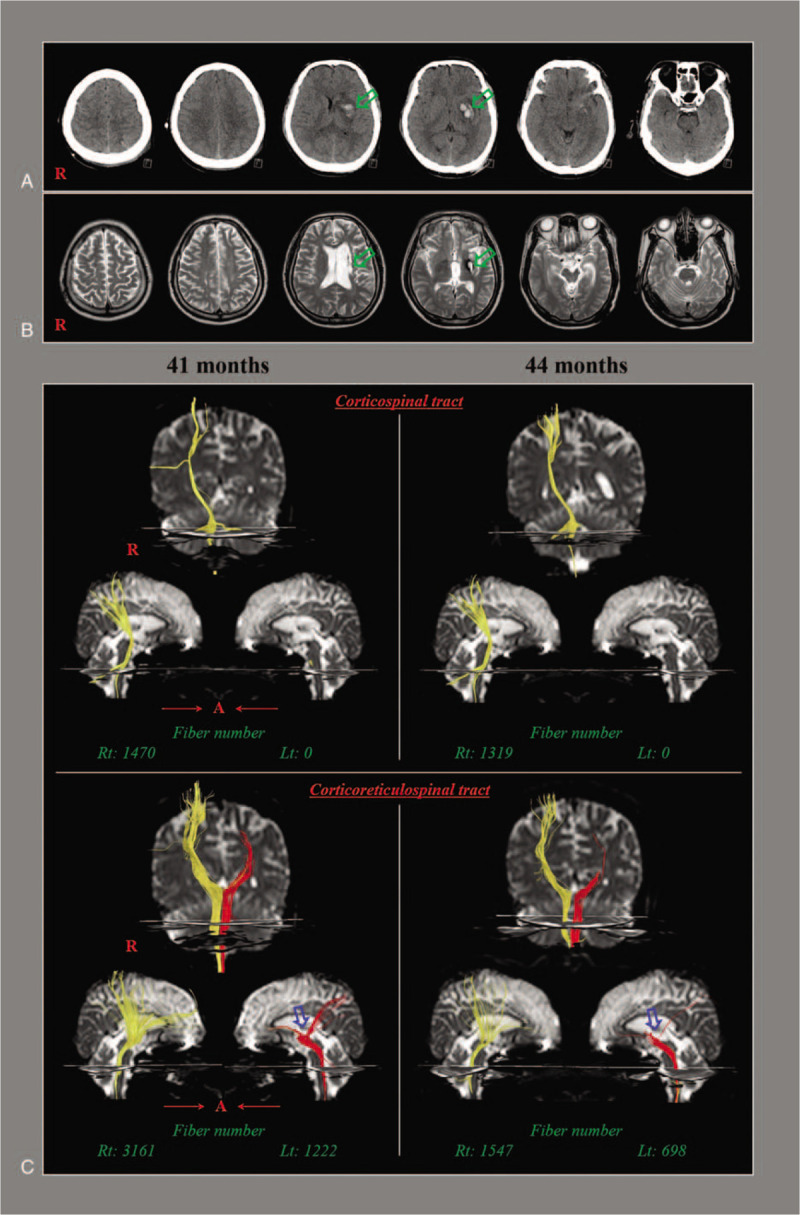
(A) Brain computed tomography at onset show traumatic intracerebral hemorrhage (arrows) in the left basal ganglia and subarchnoid hemorrhage. (B) T2-weighted brain MR images at 41 months after onset reveal leukomalactic lesions (arrows) in the left corona radiata and basal ganglia. (C) Diffusion tensor tractography (DTT) for the corticospinal tract (CST) and the corticoreticulospinal tract (CRT). The fiber number of the right CST is decreased on 44-month DTT (1319) compared with 41-month DTT (1470) and the left CST is not reconstructed on both DTTs. The fiber number of both CRTs are decreased on 44-month DTT (right: 1547, left: 698) than 41-month DTT (right: 3161, left: 1222). The left CRT shows partial tearing in the subcortical white matter (arrows) on both DTTs.

A 44-year-old male with no previous history of neurological, physical, or psychiatric illness suffered from head trauma resulting from a pedestrian-car crash: he was struck by a running taxi. He underwent conservative management for traumatic intracerebral hemorrhage in the left basal ganglia and subarchnoid hemorrhage (Fig. 1A). Following the head trauma he lost consciousness for approximately 10 days, and experienced post-traumatic amnesia for approximately 15 days. His Glasgow Coma Scale score was 3 when he arrived at the emergency room of a hospital. After 41 months, he was admitted to the rehabilitation department of a university hospital. T2-weighted MR images showed leukomalactic lesions in the left corona radiata and basal ganglia (Fig. 1B). The Medical Research Council (MRC) score was used for evaluation of motor function: 0, no contraction; 1, palpable contraction but no visible movement; 2, movement without gravity; 3, movement against gravity; 4, movement against a resistance lower than the resistance overcome by the healthy side; and 5, movement against a resistance equal to the maximum resistance overcome by the healthy side.^[14]^ He presented with right hemipareresis and could not even stand (MRC: shoulder abductor, 3; elbow flexor. 3; finger extensor, 0; hip flexor, 2^−^ [range: 30°]; knee extensor, 1; and ankle dorsiflexor, 3^−^).^[14]^ In addition, he exhibited slow, clumsy, and mutilated movements when performing movements of his right ankle. He received rehabilitative therapy including dopaminergic drugs (pramipexole 2.5 mg, ropinirole 2.5 mg, and amantadine 300 mg, and carbidopa/levodopa 75 mg/750 mg), movement therapy at sections of the physical and occupational therapies: motor strengthening of the trunk and right leg, and exercises for trunk stability and control, static and dynamic balance training on sitting and standing positions, and neuromuscular electrical stimulation of the right knee extensor and ankle dorsiflexor muscles. The right leg weakness slowly recovered during 3 months, until 44 months after the initial injury (MRC: shoulder abductor, 3; elbow flexor, 3; finger extensor, 0; hip flexor, 3; knee extensor, 3; and ankle dorsiflexor, 3^+^). As a result, he was able to walk on an even floor with holding parallel bar with his left hand. The patient provided informed consent, and the study protocol was approved by our institutional review board. The patient provided signed, informed consent and our institutional review board approved the study protocol.

### 2.1 Diffusion tensor tractography

DTI data were acquired 2 times (41 and 44 months after onset) using 6-channel head coil on a 1.5 T Philips Gyroscan Intera (Philips, Ltd., Best, the Netherlands) with single-shot echo-planar imaging. For each of the 32 noncollinear diffusion sensitizing gradients, we acquired 63 contiguous slices parallel to the anterior commissure-posterior commissure line. Imaging parameters were as follows: acquisition matrix = 96 × 96; reconstructed to matrix = 192 × 192 matrix; field of view = 240 mm × 240 mm; TR = 10,398 ms; TE = 72 ms; parallel imaging reduction factor (SENSE factor) = 2; EPI factor = 59; b = 1000 s/mm^2^; NEX = 1; and a slice thickness of 2.5 mm. The fiber assignment continuous tracking (FACT) algorithm implemented within the DTI task card software (Philips Extended MR WorkSpace 2.6.3) was used in performance of fiber tracking. For corticospinal tract (CST) analysis, a seed region of interest (ROI) was placed on the anterior blue portion of the upper pons and a target ROI was drawn in the anterior blue portion of the lower pons on an axial slice of the color map.^[15]^ For corticoreticulospinal tract (CRT) analysis, a seed ROI was placed on the reticular formation of the medulla, and a target ROI was placed on the tegmentum of the midbrain.^[16]^ The termination criteria applied were FA <0.15 and an angle change of >27 degrees.^[15,16]^

The fiber number of the right CST was decreased on 44-month diffusion tensor tractography (DTT) (1319) compared with 41-month DTT (1470) and the left CST was not reconstructed on both DTTs (Fig. 1C). The fiber number of both CRTs were decreased on 44-month DTT (right: 1547, left: 698) than 41-month DTT (right: 3161, left: 1222). The left CRT revealed partial tearing in the subcortical white matter on both DTTs.

## 3 Discussion

In this study, this patient showed significant motor recovery in the hemiparetic (right) leg without motor recovery in the hemiparetic arm during 3 month's rehabilitation at a chronic stage from 41 to 44 months after onset. We think that the motor recovery of the hemiparetic leg in this patient was mainly attributed to the improvement of LKA for the following reasons. First, the CST and CRT which are the main neural tracts for motor function showed aggravation of these tracts in both hemispheres between 41 and 44 months because the fiber numbers of these neural tracts were decreased without significant configurational recovery on 44-month DTT compared to 41-month DTT.^[2,17,18]^ Second, the patient showed severe motor weakness in the right leg with clinical characteristics of LKA (slow, clumsy, and mutilated) which were compatible with those of LKA at 41 months after onset.^[10–12]^ Third, this patient revealed significant motor recovery of the right leg during a chronic stage (during 3 months from 41 months to 44 months after onset) with comprehensive rehabilitation including dopaminergic drugs, which have been reported to be effective for improvement of LKA.^[5–7,19,20]^ The CST, the most important neural tract for motor function in the human brain, is known to be involved in total motor function of extremities; especially, the motor function of distal extremities.^[17]^ In contrast, the CRT, which is an important neural tract in motor function next to the CST in the human brain, innervates the axial muscles and the proximal muscles of the extremities, accounting for 30%∼40% of the total proximal motor function.^[2]^ As a result, we think that this patient did not reveal complete recovery of his right hemiparesis during 3 months from 41 months to 44 months after onset because the left CST (non-reconstruction) and CRT (partial tearing) which were responsible for right hemiparesis did not show significant recovery during this period.

Regarding the motor recovery due to improvement of LKA, a few case studies have reported.^[5–7]^ In 2013, Jang^[5]^ reported on a patient with thalamic hemorrhage who showed motor recovery by improvement of LKA after one month's rehabilitation during a chronic stage including dopaminergic drugs (ropinorole: 3 mg, bromocriptine: 10 mg, and levo-dopa: 375 mg). In 2016, Jang et al^[6]^ reported on a patient with a middle cerebral artery territory infarct who showed severe left hemiparesis with complete weakness of the left finger flexor and extensor. The patient showed significant motor recovery to a nearly normal state ager 2 weeks rehabilitation including dopaminergic drugs (ropinirole, 3 mg; amantadine, 300 mg; and levodopa, 500 mg) which was started at 2 weeks after onset. The injuries of the corticofugal tracts from the secondary motor area were confirmed on DTT in this patient. Recently, Jang et al^[7]^ (2018) reported on a patient with TBI who presented with motor recovery concurrent with recovery of injured CFTs after 2 month's rehabilitation including dopaminergic drugs (pramipexole: 2.5 mg, amantadine: 300 mg, ropinirole: 0.75 mg, and levodopa: 500 mg) which was started at 4 weeks after onset.

In conclusion, we report on a patient who presented with motor recovery of the hemiparetic leg by improvement of LKA at a chronic state of TBI after undergoing rehabilitation. We believe that our results have important implications for neurorehabilitation. However, because it is a case report, this study is limited. Further studies involving larger numbers of cases should be warranted.

## Author contributions

**EBC:** study concept, design, and critical revision of manuscript for intellectual content.

**JYK:** study design, and development, and critical revision

**SHJ:** study concept and design, manuscript development, writing, funding, and critical revision of manuscript for intellectual content.
